# The Black Fig Fly, *Silba adipata* (Diptera: Lonchaeidae): Current Knowledge and Future Research Needs for an Invasive Pest of Fig Production

**DOI:** 10.3390/insects17030247

**Published:** 2026-02-26

**Authors:** Rodrigo Lasa, Iain MacGowan, Julián Bartual, Trevor Williams

**Affiliations:** 1 Instituto de Ecología AC, Xalapa 91073, Mexico; trevor.williams@inecol.mx; 2 National Museum of Scotland, Edinburgh EH5 1JA, UK; imacgowan9@gmail.com; 3 Estación Experimental Agraria de Elche, Conselleria de Agricultura, Ganadería y Pesca, Servicio de Transferencia de Tecnología, 03290 Elche, Spain; bartual_jul@gva.es

**Keywords:** fig, latex, monitoring, mass trapping, control, natural enemies, invasive status

## Abstract

The presence of *Silba adipata* in countries outside its native range has raised increasing concern among fig (*Ficus carica* L.) producers due to its growing economic importance. Information on this lonchaeid remains scarce and is frequently fragmented across regional reports, technical documents, and isolated scientific studies. The lack of consolidated and reliable knowledge on *S. adipata*, together with its sporadic impacts on figs and frequent confusion with closely related species, has hindered the development of effective and sustainable management strategies. Here, we provide a comprehensive synthesis of the current geographic distribution of *S. adipata* and its expansion into new areas, addressing diagnostic aspects and potential misidentifications reported in the literature that have the potential to confound distribution records and assessments of its pest status. We critically review current knowledge of the biology, ecology, and behavior of *S. adipata*, together with existing and emerging management and control approaches, including cultural practices, monitoring tools, mass trapping strategies, and biological- and chemical-based tactics. The strengths and limitations of these approaches are discussed in the context of their applicability to fig production systems. Advancing the management of *S. adipata* requires not only accurate species identification and evaluation of conventional control methods, but also a deeper understanding of its ecology and sexual behavior. Addressing these knowledge gaps will be essential for the development of innovative, effective, and environmentally sustainable management strategies for this emerging pest.

## Simple Summary

*Silba adipata* has recently emerged as an economically important pest of fig crops in several regions outside its native distribution. Current knowledge of this species remains limited and scattered across diverse sources. In this review, we synthesize the available information on *S. adipata* with the aim of providing an integrated overview of its status as an agricultural pest. We summarize its geographical distribution, its spread beyond its presumed area of origin and examine current knowledge of the biology, ecology, and behavioral traits of *S. adipata* that are relevant to its interaction with fig production systems. Existing management and control strategies are reviewed, including cultural, chemical and biorrational approaches, highlighting their strengths and limitations. We identify key knowledge gaps and research priorities that must be addressed to improve the development of effective, sustainable, and biorational management strategies for this invasive pest.

## 1. Introduction

The fig, *Ficus carica* L. (Moraceae), native to the Middle East and Mediterranean region, is considered one of the oldest domesticated fruit crops. This plant now has a worldwide distribution owing to its ability to adapt to a wide range of climatic conditions and soil types, particularly in tropical and subtropical regions. Consequently, fig production is widespread in countries such as Turkey, Greece, Iran, Egypt, Morocco, and Spain, as well as in the Americas (e.g., USA, Mexico, Colombia, Ecuador, Peru, and Argentina) and Asia (e.g., China, India, and Japan) [1]. Botanically, the fig is a syconium, a fleshy hollow structure containing numerous tiny, inverted flowers that develop into small fruits (achenes) following pollination. Figs have attracted growing interest due to their health and nutrition properties, which include antioxidants, fiber, magnesium, potassium, calcium, and vitamins D and K [2].

Some dipteran species are among the most important pests of figs worldwide, including some tephritids, drosophilids and lonchaeids [3,4]. One of the most important pests affecting figs is the black fig fly, *Silba adipata* McAlpine, 1956 (Diptera: Lonchaeidae) [5]. Similar to its host plant, *S. adipata* is considered native to the Mediterranean basin and the Middle East. The fly was first detected in Italy by Savastano in 1915 [6], who at the time mis-identified it as *Lonchaea aristella* Becker, 1902 and referred to it colloquially as the “black fig fly”, a name still used to this day. Subsequently, biological and morphological information was provided by Silvestri [7], again using the name *L. aristella*, representing the earliest detailed study of this species. Several decades later, McAlpine [5] corrected this taxonomic error and formally described the fly as *Silba adipata*.

**Figure 1:**
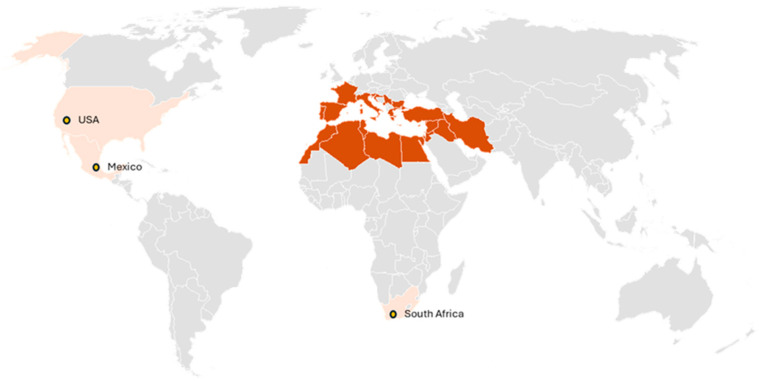
Native range of *S. adipata* (dark orange) and recently invaded areas (pale orange).

This insect has been reported throughout much of the Mediterranean region of Europe, southwestern Asia, and North Africa (Figure 1), including France, Spain (Iberian Peninsula, Canary Islands, and Balearic Islands), Portugal, Italy, Malta, Slovenia, Croatia, Montenegro, Serbia, Bulgaria, Cyprus, Greece, Republic of Türkiye, Ukraine, Syria, Jordan, Iran, Iraq, Israel, Egypt, Morocco, Tunisia, Libya and Algeria [7,8,9,10,11,12,13,14]. Outside its native range *S. adipata* was first reported as an exotic pest in South Africa in 2007 [15], in Mexico in 2020 [16], and subsequently in California, USA [17] (Figure 1). To date, there is no confirmed evidence of its establishment in Central or South America, despite the presence of fig production in these regions.

Reports of *S. adipata* in Asia east of Iran remain highly uncertain. Although Raz [18] mentioned its presence in Japan, no primary reference supports this record, and recent taxonomic reviews of *Silba* species in Japan do not include *S. adipata* [19,20]. Similarly, the occurrence of *S. adipata* infesting *Ficus variegata* Blume and *Capsicum* spp. in Indonesia remains particularly questionable. Merta et al. [21] reported genetic data for specimens identified as *S. adipata* in this country; however, the reported COI gene sequence identity (89%) falls well below commonly accepted thresholds for species-level confirmation. Moreover, these sequences were not deposited in GenBank, precluding independent verification and clarification.

Notably, the presence of several previously undescribed *Silba* species in Asia has been reported including *Silba lashker* MacGowan & Razak, 2012 from north-west India whose larvae are also found in *F. carica* [22]. *Silba ishigaki* MacGowan & Okamoto, 2013 has been found infesting *F. variegata* in Japan and Taiwan [19], *Silba ischnopoda* MacGowan & Compton, 2018 in mature male figs of the dioecious *Ficus ischnopoda* Miq. in Thailand and Cambodia [23] and *Silba capsicarum* McAlpine, 1956 on the island of Java, Indonesia [24]. Reports of *S. adipata* populations in south-west Asia are therefore likely to be misidentifications given the taxonomic complexity of the genus. This issue warrants further investigation using integrative taxonomic approaches. In contrast, *S. adipata* was conclusively detected and genetically confirmed (100% COI gene sequence identity) in shipments of figs from Turkey that were intercepted at the port of Zhuhai, Guangdong Province, China in 2018; however, to date, the species has not become established in China [25].

In light of the ongoing geographic expansion of this species and the increasing impact on fig cultivation, this review aims to provide a comprehensive synthesis of its status as an agricultural pest, critically examining current knowledge of its biology, ecology, and behavior in order to inform present and future management strategies for this invasive pest.

## 2. Invasion and Establishment in New Geographic Areas

The introduction, establishment, and subsequent spread of *S. adipata* into new geographic regions appear to follow a relatively rapid unnoticed invasion pattern, as evidenced by its detection in South Africa, Mexico, and the United States. In South Africa, Giliomee et al. [15] (2007) reported the simultaneous detection of *S. adipata* in five local municipalities along the southwestern coast, separated by several tens of kilometers. This spatial pattern suggests that the fly had been present for some time prior to detection, likely overlooked because it was initially regarded as a secondary invader of figs. This delay likely facilitated its establishment and early dispersal prior to its formal identification as an exotic pest [15] (Giliomee et al., 2007).

In Mexico, following its initial detection in central Mexico in 2020 (municipality of Ayala, Morelos State), and in accordance with the International Plant Protection Convention, a national surveillance and eradication program was implemented, initially classifying *S. adipata* as a transient actionable pest. However, once phytosanitary surveillance activities were initiated, the species was simultaneously detected in traps placed in fig orchards located in distant regions of the country, including in the States of Puebla, Aguascalientes, Coahuila, Michoacán, Hidalgo, and the State of Mexico [26]. Due to the large distances between detected sites, this pattern strongly suggests that *S. adipata* had become established in Mexico prior to its official detection and had likely begun its expansion several years earlier. The fly continues to expand its distribution across fig-producing areas of the country and was detected in 2022 in the coastal state of Veracruz, the third largest fig-producing region in Mexico [27].

A similar situation was observed in the United States, where the initial detection of *S. adipata* by employees of the California Department of Food and Agriculture was reported simultaneously in seven counties along the Pacific coast of southern California. To date, the fly has not been reported colonizing the main fig-producing areas located further north in Merced and Madera counties in California’s Central Valley [17].

The rapid invasion of *S. adipata* is noteworthy, given its strict monophagy and exclusive association with *F. carica*. Although the precise introduction pathways into South Africa and the Americas remain unknown, the movement of infested figs is considered one of the primary dispersal mechanisms and likely explains the rapid spread of this pest into fig-producing regions. As *S. adipata* preferentially infests small figs, dispersal might also occur via the transport of nursery plants that occasionally bear small, but already infested figs. In Mexico, evidence suggests that the introduction of *S. adipata* into the fig-producing region of Tatatila, Veracruz State, in 2022 occurred through the transport of used sacks from fig plantations in the neighboring state of Puebla, which unknowingly contained fly larvae or pupae [28].

## 3. Taxonomy and Identification

The black fig fly is a small, glossy dark hairy fly with a blue-black coloration, measuring approximately 3.5–4.5 mm in body length, with entirely black legs, and closed wings extending beyond the tip of the abdomen. The brick red eyes are characteristic of this species. In the Neotropics these red eyes immediately allow the observer to differentiate *S. adipata* from fig-infesting species of the lonchaeid genus *Neosilba* Waddill & Weems, 1978 which have brown eyes. Species identification is based primarily on male genital morphology, together with additional diagnostic characters that have been widely provided and illustrated in several studies [5,7,11,29,30,31,32,33]. The morphology of eggs, larvae and pupae have also been described and illustrated in detail [7,34,35,36].

Taxonomic identification within the family Lonchaeidae (Diptera) is inherently challenging due to the high degree of external morphological similarity among closely related species [11,31]. *Silba* Macquart, 1851 (Lonchaeinae: Lonchaeini) is a genus comprising some 121 described species, distributed predominantly in the Afrotropical, Indomalayan and Australasia-Oceania realms, with relatively few representatives in the Palearctic [37]. Only one rather doubtful species is attributed to this genus and two introduced species are known from the Americas where species of the sister genus *Neosilba* are predominant.

*Silba adipata* was formally described by McAlpine [5] based on a male holotype from Italy. This clarified the taxonomic confusion that had surrounded this species for several decades. *Silba adipata* had been previously mis-identified as *Lonchaea aristella* now considered to be a junior synonym of *Silba virescens* Macquart, 1851 another species common in the Mediterranean area. McAlpine [5] further noted that one of the paratype specimens of *S. adipata* had been previously described as *Lonchaea = Silba gibbosa* (de Meijere, 1910) collected in Sudan in 1915, again underlining the taxonomic difficulties in determining species of this genus.

Molecular studies of *Silba* species remain limited, which hinders reliable species-level identification using genetic tools. Currently, the GenBank database includes sequence data for only two members of the genus; *Silba fumosa* (Egger, 1862) and *S. adipata*. Although considerable sequence information is available for the mitochondrial cytochrome oxidase genes of *S. fumosa*, the available genetic information for *S. adipata* is largely restricted to the mitochondrial cytochrome oxidase subunit I (COI) gene, a maternally transmitted gene commonly used for DNA barcoding in insects [38]. Phylogenetic analysis of COI gene sequences of *S. adipata* specimens collected from figs in Israel, Turkey, and Mexico cluster within a single, well-supported genetic clade, providing strong statistical support for their conspecific status [27].

It is noteworthy that several studies from researchers in Egypt and Iraq have reported on the biology and management of *S. virescens* infesting figs [39,40,41]. This again probably has its origins in past taxonomic confusion when *Lonchaea = Silba aristella*, the junior synonym of *S. virescens*, was the name applied to *S. adipata* in earlier publications. *Silba virescens*, which has been reared on several occasions from shoots, stalks and ears of maize and from the stems of sugarcane seems to have a very different and more varied larval ecology. As *S. adipata* is considered monophagous, and in the absence of molecular data for *S. virescens*, it seems likely that the records of *S. virescens* in figs in Egypt and Iraq represent misidentifications, although specific studies are needed for clarification.

## 4. Crop Damage

**Figure 2:**
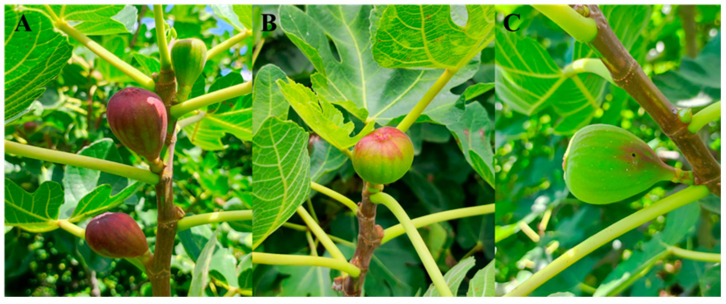
Different patterns of infested figs of the Brown Turkey variety with main reddish coloration (**A**) in the whole fig, (**B**) around the ostiole or (**C**) an infested fig with very little coloration but with an evident larval exit hole.

*Silba adipata* primarily infests small, immature figs, although mature syconia may occasionally be attacked [42], particularly after the harvest when the availability of small figs declines. The mean diameter of infested parthenocarpic figs cultivated in Mexico ranges from 2.3 to 3.0 cm in diameter (between 10 and 13 g) [27,36,43]. Infested figs can be distinguished from healthy figs, although signs of infestation vary across fig varieties and with the stage and severity of the infestation. In general, a purple discoloration is observed as a characteristic sign of larval infestation (Figure 2A,B). This discoloration may be localized on the lateral surface of the fig (Figure 2A) or concentrated around the ostiole (Figure 2B). This coloration is more evident as larval development progresses or as the number of larvae within the fig increases [27]. However, Abbes et al. [8] reported that such purple color changes are absent in fig varieties that remain green at maturity. Consistent with this observation, we have observed that no discoloration of the ‘Brown Turkey’ variety was frequent if the infestation comprised just one or two larvae (Figure 2C) [27].

Infested figs are typically abscised prematurely, resulting in substantial economic losses. The magnitude of crop losses varies widely and is largely influenced by cultivar, season, and production region. According to their pollination mechanisms, figs are divided into four types: (i) male figs known as ‘Caprifig’, which are non-edible figs that act as a source of pollen, (ii) “Smyrna” which are female figs that require pollination by the fig wasp, *Blastophaga psenes* L. (caprification) for fig production, (iii) San Pedro figs that produce brebas which are parthenogenetic figs produced in spring time and pollinated figs at the end of summer, and (iv) common figs, which are facultative parthenocarpic figs producing both unpollinated and pollinated syconia. Infestation by *S. adipata* has been documented across all fig types, including common figs, brebas, female figs, and male figs. In Tunisia, the prevalence of infestation ranges from 1% to 88%, with infestation of 70–80% or more in several breba, pollinated fig, and male fig varieties [8]. In Turkey, the prevalence of fruit drop ranges from 20% to 64% in the Smyrna cultivars ‘Sirlop’ and ‘Black Bursa’ [44]. Similarly, infestation of between 2% and 90% of the syconia has been reported in the parthenocarpic varieties ‘Black Mission’ [36,43] and ‘Brown Turkey’ [27] in Mexico.

## 5. Biology and Ecology

### 5.1. Life Cycle

**Figure 3:**
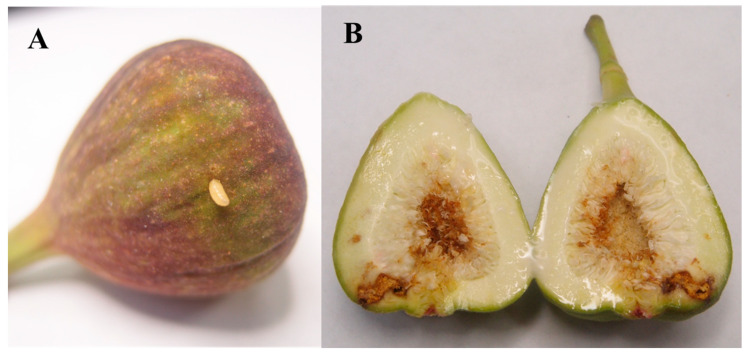
*Silba adipata* larva (**A**) exiting the fig, and (**B**) larval feeding results in brown galleries with larval frass in infested figs.

The life cycle of *S. adipata* begins when the female alights on a fig, locates the ostiole, and inserts her ovipositor to deposit eggs in small, concealed clusters beneath the ostiolar scales. The number of eggs laid is influenced by fig availability but typically varies between 1 and 8 eggs per fig and only exceeds this when the availability of oviposition sites is limited [27,36,42]. Exceptionally, a maximum of 22 and 63 pupae from a single fig, probably from different females, were reported by Lasa et al. [27] and Katsoyannos [42], respectively. Eggs hatch within three to eight days [7] and the larvae excavate the tissues of the syconium, initially feeding around the ostiole and at the base of the receptacle (pulp) before feeding within the remainder of the fig. Larval feeding galleries are typically brown, large, and irregular in shape, and frequently contain larval frass (Figure 3). This feeding activity commonly induces premature abscission of infested syconia, with only a small proportion of infested figs remaining attached to the tree [8]. Upon reaching their full development, larvae exit the fig to pupate in the soil, leaving conspicuous exit holes, even in figs that remain attached to the tree (Figure 2C). The larval exit hole is usually located in the central-lower part of the fig (Figure 3A), although occasionally it can be found near the pedunculus [27]. Larval development takes place entirely within the fig, lasting approximately 22–29 days in spring and 7–22 days in summer [34,36]. Pupation within the syconium has been reported only rarely [36,42].

Larvae of this species usually move by curling into a loop and adopting a leaping behavior, similar to that described for the Mediterranean fruit fly *Ceratitis capitata* (Wiedemann, 1824) (Diptera: Tephritidae) [45]. Larvae burrow into the soil to pupate. For *S. virescens*, pupation depths of 2–4 cm, and occasionally 5–6 cm, have been reported across different soil types. Pupae experimentally buried at depths outside this range, or in soils with high salinity or excessive moisture, experienced high mortality [39] (Abdullah and Al-Azawi 1992). The pupal stage lasts approximately 9–10 days and appears to be of similar duration during the spring and summer [36,42], but can increase to 16 days in the autumn [34]. As with most insects, the overall duration of the life cycle, from egg to adult, is temperature-dependent and generally ranges from 18 to 25 days under favorable climatic conditions and 40–50 days at cooler temperatures [7,34,42]. Although comparative studies on the fly’s developmental duration across different cultivars are lacking, the mean time to adult emergence from infested figs collected in the field (following natural oviposition), and maintained under similar laboratory conditions (24–25 °C), ranged from 17 to 19 days in the ‘Brown Turkey’ cultivar [25] and 24–26 days in ‘Black Mission’ [36].

**Figure 4:**
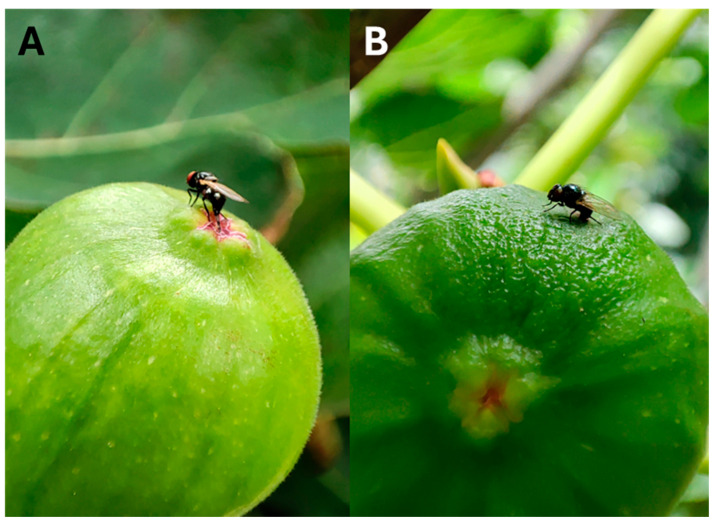
Oviposition behavior of (**A**) *Silba adipata* at the ostiole and (**B**) *Neosilba* sp. in a larval exit hole left by previous *S. adipata* infestation of figs in Veracruz, Mexico.

It is important to mention that the larval exit holes of *S. adipata* could be utilized by opportunistic species of lonchaeids for their development. In contrast to *S. adipata* oviposition in the ostiole (Figure 4A), *Neosilba* spp. females have been observed ovipositing inside the exit holes of *S. adipata* larvae (Figure 4B). In Mexico, several *Neosilba* species, including *Neosilba batesi* (Curran, 1932), *Neosilba glaberrima* (Wiedemann, 1830) *Neosilba flavitarsis* MacGowan and Lasa, 2025 and *Neosilba recurva* MacGowan and Lasa, 2025 were registered emerging from fruits previously infested by *S. adipata* [27,46]. The life cycle of these *Neosilba* species has not been studied but adults emerged about 10–15 days later than *S. adipata*, a temporal lag related to the later infestation of figs by the *Neosilba* species.

In Europe, *S. adipata* is thought to overwinter primarily as pupae in the soil [47], with adult emergence occurring in the early spring. The lower lethal temperature for overwintering stages has not been experimentally determined, but available evidence suggests that the pupae can tolerate winter temperatures slightly below −3 °C, although a persistent cold period could significantly reduce springtime populations [34]. *Silba adipata* has been reported from lowland regions ranging from sea level up to approximately 1000 m a.s.l. in parts of Europe and in California (USA), whereas in Mexico the fly is largely restricted to fig-producing areas at higher elevations, typically around 1800–2000 m a.s.l.

Adult longevity in *S. adipata* can exceed 6 weeks under controlled conditions. Laboratory adult longevity was greater for females (98 days) than for males (78 days) in flies reared from ‘Brown Turkey’ figs [48] which was longer than reported for flies reared from the ‘Black Mission’ variety (45–55 days for both sexes) [36]. Based on the development of the reproductive organs, males appear to reach sexual maturity within a few days, whereas females require at least 2–3 weeks to achieve sexual maturity, and only do so when protein is consumed during this period [48]. Females that consumed only carbohydrates failed to achieve sexual maturity, as indicated by their ovarian development, which remained almost unchanged from that observed at emergence. Females exhibit an ovarian load of approximately 20–24 eggs [48], which is considerably lower than that (>1000 eggs) reported for many tephritids [49]. Protein intake required for sexual maturation has been a fundamental biological trait exploited in the management of tephritid pests, forming the basis for the development of monitoring attractants and control strategies such as mass trapping and toxic bait applications [50,51,52]. It seems likely that similar approaches may be applied to the monitoring and control of *S. adipata* (see Section 6).

### 5.2. Feeding, Mating and Oviposition Behavior

The activity of *S. adipata* adult flies appears to follow a bimodal daily pattern, with two main peaks—one at dawn, before incident sunlight reaches the canopy, and a second at dusk [34,42]. According to Katsoyannos [42], fly activity initiates at dawn at temperatures above 18 °C. We have observed similar activity patterns in greenhouse experiments in which fly activity is concentrated during the early and mid-morning (07:00–11:00 h) and declines sharply as temperatures increase beyond 25–26 °C or incident sunlight exceeds 5000 lux, followed by an additional period of activity in the late afternoon (15:00–17:00 h) approximately 1–2 h before dusk [53].

Adults have been reported to feed on the juice exuding from overripe figs attached to the trees, and more frequently on droplets of fresh or dried milky fig-tree sap that is exuded when unripe figs or leaves are injured [42]. In the absence of overripe figs, adults have been observed feeding on the exudates of fig wax scales and other insects and have been observed visiting ornamental flowers or around fruit crops [42]. As mentioned above, fig latex has been reported to be highly attractive to this pest [34,36,42,48]. A highly effective method for detecting *S. adipata* involves releasing latex by breaking tender shoots of the fig plant [42]. Groups of five or more adult flies may aggregate to feed on latex droplets, with no aggressive interactions observed despite close physical proximity during feeding [34].

Latex remains attractive even after drying, up to one day after its release [42]. Both males and females are attracted to fresh latex droplets, with longer-lived females exhibiting higher responsiveness and greater latex consumption at the onset of sexual maturity (aged 20 days). Latex is a complex aqueous suspension of secondary metabolites comprising terpenoids, alkaloids, tannins, phytosterols, enzymes and proteins present in fruits, leaves and stems, that is secreted in appreciable quantities to protect the plant against external threats [54]. The reasons for attraction to latex in *S. adipata* are unclear. Flies deprived of protein showed increased attraction to latex compared to flies fed a protein-rich diet [48]. However, despite containing some amino acids (cysteine, leucine, ornithine, serine, tryptophan, and tyrosine), latex has a low overall protein content [55], suggesting that attraction to latex is not primarily related to protein requirements. In some tephritid species, the consumption of highly attractive plant-specific compounds has been associated with enhanced sexual behavior and stimulation of oviposition [56,57].

Despite frequent observations of *S. adipata* adults feeding on carbohydrate-rich substances, there are no reports of protein consumption by this pest in the field, even though protein intake is required for sexual maturation. In natural environments, protein sources are often scarce, and other dipteran species have been reported to acquire proteins from bird feces [58], pollen [59] or from microbial communities [60].

The movement of adults in the crop canopy has been noted but has not been studied in detail. Adult flies exhibit rapid, zig-zag flight with unpredictable trajectories, followed by a marked deceleration immediately before landing [34,42]. The presence of many flies in the same area, which can be observed on consecutive days, suggests some gregarious behavior for this species [34]. Further studies are required to confirm this pattern, particularly given its potential implications for the efficacy of monitoring and control strategies. In the field, detection is facilitated by inspecting the outer canopy leaves in shaded or partially shaded areas, especially on young shoots and tender leaves, where adults are commonly found on the abaxial leaf surface, presumably to avoid direct solar radiation [34].

The mating behavior of this species remains largely unknown, although as in other lonchaeid species, it is considered likely to involve adult swarming behavior often carried out in sunny, sheltered glades several meters above ground level [61]. As reported by Katsoyannos [42] and confirmed through our three years of field observations and laboratory rearing of *S. adipata* from infested figs, no mating activity was observed either in the field or under cage conditions in the laboratory, even when stimuli such as ripe or unripe figs and leaves were introduced into the cages. To date, only a single mating swarm has been documented in the field for *S. adipata* [62], and only one copulating pair has been reported and photographed [34]. Understanding the mating behavior of this species is a key step in our ability to continuously rear the fly in controlled laboratory or greenhouse conditions, which would greatly facilitate detailed studies on the biology and behavior of this species.

Female flies have been observed ovipositing throughout the day, but mostly during the afternoon and until dusk [42]. After a characteristic zig-zag flight, females land on the fig, locate the ostiole and insert the ovipositor between the surrounding scales. Oviposition involves brief probing movements, after which females walk around the ostiole to lay additional eggs on the same fig, typically changing orientation by 90–180° between successive bouts of egg laying [42]. This sequence is repeated multiple times, resulting in eggs being deposited in small, closely spaced clusters beneath the scales of the ostiole.

To date, there are no confirmed records of infestation on host plants other than *F. carica*. The paratype adult reportedly collected from eggplant (*Solanum melongena* L.) [5] is a female specimen and, lacking the clear specific characters found in the male genitalia, its identity is questionable until established by DNA sequencing. More recently, the occurrence of *S. adipata* in chili peppers has been reported in Indonesia [63,64]. However, these records also remain questionable, as they most likely correspond to *S. capsicarum*, a morphologically similar species previously described infesting peppers on the island of Java, Indonesia [24]. The low genetic similarity (89% COI sequence identity) reported by Merta et al. [19] supports the notion that the specimens from peppers are not *S. adipata*.

Anecdotal reports indicate an oviposition preference for some specific fig varieties, although no systematic comparisons have been published. In other species of fruit flies, oviposition preference can be related to fruit size, color, firmness, ripeness, skin texture, ostiole width and structure, water content, chemical composition and host-associated microbes [8,65,66]. A significant reduction in oviposition was observed by *S. adipata* in pollinated compared to unpollinated figs in the facultatively parthenocarpic “Brown Turkey” variety [67]. This was correlated with variation in organic volatile compounds emitted by pollinated and unpollinated figs with some compounds (geranyl acetone, hydrocinnamaldehyde, α-thujene, terpinene-4-ol, δ-elemene and α-terpineol) only present in unpollinated figs. These findings suggest that the application or manipulation of specific compounds could potentially reduce oviposition levels, an aspect that warrants further investigation studies.

### 5.3. Population Dynamics

Although information from different geographic regions remains limited, *S. adipata* is generally considered to complete four to six generations per year [27,34,68]. The population dynamics appear broadly consistent across regions, with pronounced population increases occurring in mid-summer, particularly in July and August [18,27]. Following the winter period, population densities are usually very low and require two to three generations to rebuild. As temperatures increase, adult activity resumes, with the first detections of adults reported in late February in southern Spain [69] and during the first half of March in southwestern France [34] and Veracruz, Mexico [70]. The initial detections of damaged figs are commonly observed in April, which are likely caused by adults that emerged in March [7]. The last adult detections in the field tend to occur in November and December, when figs are no longer available [27,34,69]. Nevertheless, in central Mexico, where pruning practices promote substantial autumn fig production, *S. adipata* has been reported infesting figs during the winter period, from November to February when diurnal and nocturnal temperatures are typically around 22 and 2 °C, respectively [32,71].

## 6. Monitoring and the Response to Traps and Attractants

**Figure 5:**
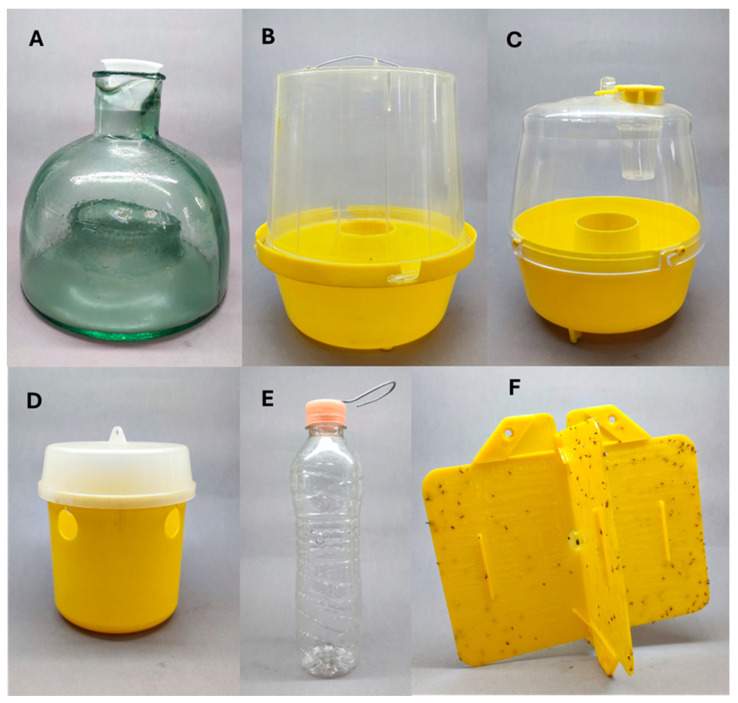
Different model traps used to monitor *S. adipata*: (**A**) glass McPhail, (**B**) plastic McPhail, (**C**) Multilure, (**D**) Tephri trap, (**E**) handmade bottle trap and (**F**) Rebell sticky trap.

Attractant type and trap color have been among the most important factors considered for improving trap efficacy and specificity in many tephritid fruit flies [72], whereas trap and attractant combinations have been addressed in only a few studies involving Lonchaeidae. Monitoring of *S. adipata*, has mainly been performed using liquid attractants in traps previously developed for tephritids such as the glass McPhail trap (Figure 5A) [42], the plastic McPhail trap with a yellow base (Figure 5B) [34], the Multilure trap (Figure 5C) [73], the Tephri trap (Figure 5D) [74,75], or cheap handmade traps constructed from modified plastic (PET) bottles with three or four holes of 0.5–0.6 mm in the side of each bottle (Figure 5E) [76,77]. Modified cups of 1 L, with different variations in the access holes, have also been proposed [43,73]. In contrast to liquid traps, visual sticky traps have not been widely used for this pest, probably due to weak response to colors and the low efficacy previously reported by a yellow Rebell^®^ sticky trap, when compared with ammonium-baited traps (Figure 5F) [42].

Laboratory assays that tested a range of colors (red, orange, yellow, blue, violet, white, and black) revealed no significant differences in fly attraction, although marginally higher capture rates were recorded for orange and yellow. Similarly, under field conditions, yellow and orange colors applied to the lower section of plastic bottle traps did not improve trap performance compared with white or transparent traps when baited with torula yeast [76].

Food attractants based on ammonium salts such as ammonium sulfate, diammonium phosphate and ammonium acetate at 2% and 4% (wt/wt) concentration have been employed for *S. adipata* monitoring [34,42,75,76,77]. Gaseous ammonia released by these traps may be used by flies as an indicator of protein availability, which is essential for sexual maturation of *S. adipata* females, as has also been reported for other tephritid flies [50,78,79]. A 2% ammonium sulfate solution was as effective as a 4% solution, but slightly more effective than 4% diammonium phosphate solution [77]. Under laboratory conditions, ammonium sulfate was more attractive than ammonium acetate when tested at equivalent concentrations, although attraction levels were similar when the concentration of ammonium acetate was reduced. We previously postulated that the lower attraction to higher concentrations of ammonium acetate could be related to the production of small amounts of acetic acid from the acetate component of the solution, which may be repellent to *S. adipata* [76]. Further experiments are required to confirm this hypothesis given that the response of this fly to fermented products that release acetic acid, is, in general, weak. There is also evidence that some fruit flies avoid acidic environments, as they do not associate them with viable food or protein sources [80].

In a study on the Mediterranean fruit fly, the capture of *S. adipata* adults was reported in a dry trap baited with Biolure^®^, a blend of ammonium acetate, putrescine and methylamine but with low captures when compared to hydrolyzed proteins [74]. In laboratory assays evaluating individual or combined organic compounds (hexanol, hexanal, hexyl acetate, heptanal, octanal, nonanol, and nonanal), only hexanol elicited a consistently high attractive response in *S. adipata* [68]. Subsequent field studies showed that hexanol alone was not more effective than a 2% ammonium sulfate solution in the capture of flies, but the combination of 2% ammonium sulfate solution with hexanol increased captures by approximately 56% compared with ammonium sulfate solution alone. In contrast, a field study in central Mexico indicated that the mixture of ammonium sulfate and hexanol was not more effective than ammonium sulfate alone [77].

Due to the strong behavioral response of *Silba adipata* to fig latex, this substance has been recommended for field monitoring in an equal-parts mixture of fig branch extract and 0.3% ammonium sulfate solution [81]. Under field conditions, adult captures of *S. adipata* were approximately twice as high when latex was provided in an aqueous solution compared to its undiluted application on a cotton pad [42]. Two independent studies demonstrated that 2% ammonium sulfate solution supplemented with approximately 0.5–1 mL (ca. 10 drops) of fig latex was significantly more effective than ammonium sulfate solution alone [42,76]. Torula yeast tablets that are pelletized with borax were also a suitable attractant [76]. A major advantage of ammonium sulfate plus latex-based attractant mixtures under field conditions was the high specificity of the traps, reflected in the low capture of non-target insects and slightly lower capture of other lonchaeid species (*Neosilba* and *Lonchaea*), at least when compared with torula yeast + borax pellets [76].

Other food-based attractants widely used for tephritid flies have also been evaluated for *S. adipata*, including commercial protein hydrolysates, such as acid-hydrolyzed proteins (e.g., Buminal^®^, Atralat^®^, Captor 300^®^) and enzymatically hydrolyzed proteins such as CeraTrap^®^ [43,75,76,77,82]. Acid-hydrolyzed proteins are commonly used in combination with borax following standard recommendations for tephritid pests [83] or have been tested with additives such as black molasses [82].

In Black mission fields, the capture of torula yeast + borax pellets was also superior to Atralat^®^, a hydrolyzed protein prepared with borax [43]. Experiments in Portugal indicated that traps containing a combination of 5% hydrolyzed protein, the tips of fig branches (3 cm long) and hexanol captured five-fold more adults than 5% diammonium phosphate solution with the same mixture of fig branches and hexanol [75]. One experiment also tested GF-120^®^ (Dow AgroSciences), a spinosad bait lure based on proteins, sugars and 1% ammonium acetate, although this attractant had a lower capture when compared with 2% ammonium sulfate solution [77].

Hydrolyzed proteins combined with borax have also been used in McPhail traps to monitor the pitaya flower-bud fly, *Dasiops saltans* Townsend (Diptera: Lonchaeidae). The highest captures were obtained with maize hydrolyzed protein; however, specificity toward the lonchaeid was also low, as other *Lonchaea* and *Neosilba* species were frequently captured [84]. Ceratrap^®^ and torula yeast pellets also captured high numbers of *Lonchaea*, *Neosilba* and *Dasiops* species [85].

Various homemade attractants, including fermented figs, fermented pineapple and pineapple juice have also been evaluated. Low overall capture rates and some inconsistency has been observed among experiments. In general, fermented attractants have low capture efficacy when compared to hexanol or torula yeast + borax pellets [43]. However, experiments conducted in autumn and winter, although characterized by low capture numbers, revealed that fermented ripe and unripe figs were more effective for detecting *S. adipata* than ammonium sulfate, torula yeast + borax pellets, or hydrolyzed protein [71]. In this case, the likely reduced availability of suitable figs, together with lower volatile emissions from torula-based traps and hydrolyzed protein under low temperatures, may have influenced the observed results.

Captures in most of the attractants, including ammonium sulfate, torula yeast + borax pellets, hydrolyzed protein + borax, hexanol, ammonium sulfate + hexanol, latex, and ammonium sulfate + latex, were female-biased, with females representing approximately 57–70% of the total captures [42,68,76,77]. In one experiment using latex as an attractant, the proportion of females increased to nearly 80% [42]. However, sex-specific responses varied seasonally, as captures were not female-biased during an experiment conducted in the rainy season using several attractants, including ammonium sulfate, torula yeast, hydrolyzed protein + borax, and ammonium sulfate + latex [76]. The physiological status of females has been less extensively studied; however, food-based attractants such as torula yeast + borax pellets and ammonium sulfate + fig latex have been reported to capture 76–90% immature females in Brown Turkey fig plantations [76], a finding that could have implications for the use of control tools targeted at females.

It is worth noting that many of the published studies lack a statistically robust experimental design involving adequate replication and trap rotation procedures. Also, these studies often report very low capture rates, so their results should be interpreted with obvious caution. In some cases, certain attractants (e.g., 2% ammonium sulfate, hexanol and torula yeast) have failed to capture adults in monitoring experiments, despite the confirmed presence of the pest in the orchard [86,87]. Consequently, robust conclusions regarding the comparative efficacy of some attractants remain difficult to establish, highlighting the need for further well-replicated and statistically robust studies conducted under laboratory and field conditions.

## 7. Control Strategies

### 7.1. Cultural Control

Of the cultural pest control practices available to growers, orchard sanitation through the systematic removal of infested figs, whether dropped on the ground or still attached to the plant, is considered one of the most effective measures for reducing pest pressure. In lonchaeids, which pupate in the soil near the host plant, unharvested and fallen figs can sustain local populations and promote continuous reinfestation. Therefore, infested figs must be properly destroyed, either by burial (~40 cm depth) or by exposure to solar heating in sealed black plastic bags to prevent adult emergence, as recommended for tephritid flies [72,88].

Abbes et al. [8] suggested the use of fruit bagging to reduce damage, although evidence supporting its application in fig production is still lacking. As fruit bagging is labor-intensive and costly, it may be a viable option for small-scale systems in countries with low labor costs or high-value production, such as fresh figs intended for export markets. When applied at early fruit developmental stages, bags act as a physical barrier that prevents oviposition. Fruit bagging cannot be used in caprifig or caprification-dependent fig varieties that require the entry of the pollinating wasp *B. psenes* for obvious reasons. However, nets with a mesh size of 2–2.5 mm may represent a feasible alternative, provided that the pollinating wasp can still enter the syconia covered by the net [8]. Further research is required to identify the most suitable bag or net materials as figs must be protected from *S. adipata* oviposition when they are still very small (~1.5–2.0 cm in diameter). Fruit bagging has been shown to significantly reduce the prevalence of infestation in several fruit fly systems and is compatible with organic and low-input production schemes [89].

Protected fig cultivation is gaining interest in order to extend fig production areas and to produce figs out of season [90]. In other regions, fig production under mesh coverings becomes necessary due to the lack of registered insecticides for the control of *S. adipata* and *C. capitata*, which can cause substantial losses [91]. Under greenhouse production, the use of double-door entry systems and the prompt repair of any openings in the mesh can substantially reduce pest entry and subsequent infestation. However, mite populations can become problematic in greenhouse production systems [92].

### 7.2. Chemical Control

Despite a paucity of studies using modern active ingredients, it appears clear that the application of broad-spectrum products often fails to provide useful levels of control of *S. adipata* [92]. This is because larvae are protected from spray applications inside the developing figs, so that the only stages vulnerable to chemical control are the adults, the final instar larvae as they exit the fig and the pupae in the soil.

Despite this, regular applications of the organophosphate triazophos reduced adult populations in Egypt [82] and current recommendations in Mexico include spray applications of diazinon when the figs are at a development stage of 20–30 days [93]. The organophosphates methidathion and dichlorvos were reported to provide effective control against this pest in Israel [18], whereas in France the only product approved for *S. adipata* control by direct application to fig plants is the pyrethroid deltamethrin, which is considered effective at reducing crop losses to this pest [34]. Others report Mexican growers applying spinosad, bifenthrin and diazinon alone or in mixtures at weekly intervals with generally unsatisfactory results [36]. Furthermore, the application of broad-spectrum insecticides in male fig varieties or in pollination-dependent cultivars should be carefully evaluated, given the potentially severe adverse effects on the pollinating wasp *B. psenes* [8].

Recent laboratory studies have highlighted the toxicity of spinosad and emamectin benzoate, two selective biorational insecticides derived from soil bacteria, against *S. adipata* adults [94]. If validated in field testing, these products could have potential for the development of toxic baits against this fly.

Previous laboratory studies indicated that soil applications of spinosad markedly reduced pupation, adult emergence and adult survival time in the tephritid *Anastrepha ludens* Loew, 1873 which also pupates in the soil [95]. Similarly, soil treatments using diazinon or fenvalerate provided control of *S. virescens* in fig plantations in Iraq [96]. Given these findings, it would be worthwhile field-testing soil treatments using modern biorational products against *S. adipata*.

The low effectiveness of spray applications against adults is also reflected in the historical literature involving the application of organophosphate and pyrethroid compounds to crops such as cassava [97,98], passionfruit [99], and peppers [100,101] against other species of lonchaeid pests. Botanical extracts of the Chinaberry tree, *Melia azedarach* (Meliaceae), have also demonstrated some insecticidal activity against other lonchaeid species, but with low efficacy [102].

For these reasons, integrated pest management strategies based on monitoring, cultural control, natural enemy conservation and the use of selective toxic baits are likely to prove more effective than standard chemical control approaches.

### 7.3. Biological Control

#### 7.3.1. Predators

There is an almost complete absence of information on the natural enemies of *S. adipata* and a marked paucity of information from other lonchaeid species, which contrasts with a diversity of studies on tephritid flies. As lonchaeids and tephritids most probably share a number of enemies in common, we consider these likely to be of greatest importance as natural agents of mortality in *S. adipata* populations.

The principal vertebrate predator of *S. adipata* adults is likely to be insectivorous birds, whereas various invertebrate predators are expected to consume the adult flies, including spiders, dragonflies and predatory flies (Asilidae). For the most part, larvae have a physical refuge from predation during their development, and are only exposed to predators as they exit the syconium to pupate in the soil. As a result, final instar larvae and pupae risk predation from ants and predatory beetles (e.g., Carabidae, Staphylinidae). Ants may also remove *S. adipata* eggs from the ostiole or attack larvae while they are abandoning the fig or after falling to the ground to pupate [34]. Studies on tephritids have highlighted the importance of ants in the predation of pupae, including species in the genera *Pheidole*, *Solenopsis*, *Pachycondyla*, *Pogonomyrmex* and *Tapinoma* [103,104,105,106]. The soil dwelling stages of tephritids are more susceptible to ant predation when closer to the surface, when soil is drier and less compacted and when in close proximity to ant nests [107,108]. A similar range of natural enemies were reported for *Dasiops inedulis* Steyskal, 1980, a lonchaeid pest of passionfruit in Colombia, with ants and ground beetles the major predators in soil and spiders, vespid wasps and chrysopids (Neuroptera) the most abundant predators on host plants [109]. Similar findings are likely to hold true for *S. adipata* stages on plants and in the soil.

#### 7.3.2. Parasitoids

The only parasitoid reported from *S. adipata* is the pteromalid *Pachycrepoideus vindemmiae* (Rondani, 1875), [42] which is a generalist endoparasitoid of pupae that also parasitizes tephritids, drosophilids and other species [110]. This species is also a facultative hyperparasitoid, so its use as a biocontrol agent has generated concerns over its ability to parasitize primary parasitoids [111]. Reports of parasitism of *S. adipata* in pepper crops in Indonesia [64,112] are likely to involve misidentification of the host as COI gene sequences indicated that the pepper pest was not closely related to *S. adipata* [21].

Despite rearing thousands of field-collected specimens of *S. adipata* from figs in Mexico, we have never observed parasitism [70]. Similarly, studies on this pest in South Africa reported the absence of parasitoids in field-collected figs infested by this fly [15]. In contrast, lonchaeids in other agricultural systems can be attacked by a diversity of parasitoids, although the prevalence of parasitism tends to be highly variable [113,114,115,116].

We believe that the absence of larval parasitoids of *S. adipata* is due to the protective effects of fig latex, which likely repels probing by the parasitoid’s ovipositor, resulting in the rapid termination of attempts at parasitism, even in species such as the braconid *Diachasmimorpha longicaudata* (Ashmead, 1905) that efficiently parasitizes tephritid larvae in infested fruit.

It is possible that soil-dwelling pupae of *S. adipata* may be susceptible to parasitism from several parasitoids that naturally attack tephritid pupae in soil, including species such as *Eurytoma sivinskii* Gates and Grissell 2004 (Eurytomidae), *Dirhinus giffardii* Silvestri, 1919 (Chalcididae), *Melittobia digitata* Dahms, 1984 (Eulophidae), *Coptera haywardi* (Ogloblin) and *Trichopria anastrephae* (Perkins, 1910) (Diapriidae) [117,118,119,120,121]. Detection of these parasitoids in *S. adipata* would involve sampling pupal populations in soil, an activity that has not be reported in the literature at present.

It is important to bear in mind that the abundance of invertebrate natural enemies and their functional importance as predators and parasitoids, will be highly dependent on the dose rate, frequency of application and class of pesticides used in fig production systems.

#### 7.3.3. Pathogens

There are no records of pathogenic viruses isolated from *S. adipata* and there is currently no evidence that the pest can be controlled through the use of bacterial bioinsecticides, such as those based on *Bacillus thuringiensis* subsp. *israelensis* or *Lysinibacillus sphaericus*. As these types of products require ingestion of the bacterial pathogen, the protected feeding of larvae is likely to prevent the larvae from consuming lethal doses of these pathogens. Greater hope lies in the use of fungal and nematode entomopathogens that have shown promise in the control of tephritid pests.

To date, no fungal pathogens have been reported for *S. adipata*. Fungal pathogens such as *Beauveria bassiana* or *Metarhizium anisopliae* infect their hosts when conidia land on the host cuticle, germinate and penetrate the integument. Consequently, fungi can be used against dipterans in four main approaches: (i) As direct contact sprays onto the insects or the host plant on which they forage [122]; (ii) By placing conidia in powder formulations in bait stations in which flies become contaminated and subsequently transmit the conidia to other flies during mating, swarming or aggressive territorial contacts with conspecifics [123,124]; (iii) By applying conidia to soil beneath host plants so that burrowing larvae, pupae and emerging adults are contaminated by conidia that subsequently infect them [125,126]; (iv) By exploiting the endophytic habits of these pathogens as an alternative route for lethal infection of the pest [127].

Similarly, entomopathogenic nematodes, particularly species of *Steinernema* and *Heterorhabditis*, have demonstrated effectiveness against tephritids when applied to soil [128]. The soil textural characteristics, moisture and depth of the dipteran pupae influence the ability of the infective juvenile stages of the nematodes to locate and infect their hosts [129,130]. In this regard, it is surprising that *S. adipata* has failed to attract the interest of the insect pathologist community given the significant advances that have been achieved in the control of tephritid pests using entomopathogenic fungi and nematodes.

## 8. Mass Trapping

Mass-trapping systems for lonchaeids currently rely on food-based attractants (Section 6). The efficacy of mass-trapping depends not only on the attractant formulation but also on several operational factors, including trap density, spatial distribution, and lure replacement frequency. High trap densities are generally required to achieve population-level impacts, particularly in orchards with a history of infestation [52]. In Mexico, the deployment of 20 handmade PET bottle traps (~600 mL capacity) per hectare, baited with three torula yeast + borax pellets dissolved in 250 mL of water and placed in shaded areas at 15 m intervals, is recommended by the government phytosanitary authority to reduce *S. adipata* infestation [73]. Torula yeast + borax pellets degrade over time, particularly under warm field conditions. As a result, these attractants typically remain effective for only two to three weeks [72,83] and require regular maintenance to ensure consistent attraction and capture rates.

Recently, the dry FlyPack Ficus trap (SEDQ Healthy Crops S.L.), incorporating 0.015 g of deltamethrin impregnated in the trap cap, has been used in Spain in combination with an ammonium salt and hexanol packaged in separate long-release sachets, although information on its efficacy is not yet available. In this region, mass trapping deployment is recommended in June using a density of 80 traps per hectare that reportedly remains effective for up to 120 days [34]. The FlyPack Ficus trap is currently undergoing the registration process in France [131].

Quantitative information on the effectiveness of mass-trapping against *S. adipata* is lacking, but observations suggest that this approach could be very effective. Traps capture both sexes, with a higher proportion of immature females in the case of food attractants, which represents a key advantage for population suppression as females are trapped before they have begun to oviposit in the crop. For optimal efficacy, traps should be deployed early in the season, if possible, prior to the onset of fruit susceptibility in spring with the aim of intercepting the founder females and to reduce local population growth early in the season. Mass-trapping is a promising control strategy that can be improved with better attractants, but it is most effective when combined with complementary control tactics, particularly orchard sanitation.

## 9. Conclusions and Future Research Needs

The increasing economic relevance and invasive characteristics of *S. adipata* as a pest of fig production outside its native range highlight the urgent need for the development of effective and sustainable management strategies. Despite recent advances, current control options remain limited and are largely extrapolated from other fruit fly systems, underscoring substantial knowledge gaps that constrain the implementation of biorational, pest-specific approaches. Addressing these gaps requires a comprehensive understanding of the fly’s biology, behavior, and ecological interactions across different types of fig production systems, as well as a deeper evaluation of potential control strategies. The accurate taxonomic identification of the pest is essential to avoid confusion with morphologically similar species.

Understanding the biology and ecology of the fly is fundamental to the design of effective pest management programs. In particular, the fly’s mating behavior is a key void in our current understanding. Information on the conditions required by *S. adipata* to facilitate mating could provide valuable insights for the establishment of laboratory colonies under controlled conditions, which would expedite biological studies and the testing of effective control tools. These include the range of modern biological and selective insecticides, molecular approaches such as the use of RNAi [132], novel genetic control mechanisms [133], the role of the insect microbiome [134], and the possible characterization of species-specific pheromones or other tools related to the fly’s chemical ecology [135], all of which are promising avenues of research in the control of tephritid pests. Studies addressing the spatial dynamics of *S. adipata* populations within fig orchards, including their relationships with microhabitat conditions and potential aggregation mechanisms, are also necessary to identify areas of high vulnerability, allowing traps and other control tools to be used to greatest effect.

Accurate pest detection and population surveillance, together with an understanding of the spatial and temporal dynamics of pest populations within crops, are fundamental components of integrated pest management (IPM) strategies [136]. The effectiveness of these approaches depends largely on the reliability and reproducibility of pest responses to trap attractants, as well as on the selectivity of these attractants toward the target species and their minimal impact on non-target insects. Despite recent advances, many older studies on *S. adipata* attractants lack rigorous experimental designs, making robust conclusions regarding the efficacy of some lures difficult to establish. Current attractants used for lonchaeid flies are generally broad-spectrum food lures, which often show low selectivity and attract a wide range of non-target insects. Future research should focus on identifying and exploiting host-specific or host-associated semiochemicals, such as latex-derived compounds and hexanol, that mediate attraction in *S. adipata*. Advanced analytical approaches, including electroantennography coupled with gas chromatography or mass spectrometry, offer powerful tools to identify biologically relevant compounds. Further optimization should target methods to improve lure stability, longevity, and release rates under field conditions.

The susceptibility of *S. adipata* to pesticides has been poorly studied, likely due to difficulties in rearing this species under controlled conditions for laboratory toxicity tests. To date, no comprehensive information has been published on insecticide efficacy against this pest. Among the potential chemical control tactics, the use of toxic baits represents a particularly promising approach but remains largely unexplored. The relatively long pre-oviposition period of *S. adipata* offers a valuable window of opportunity to target adult females before egg laying occurs. This suggests that bait-based strategies combining attractants such as protein sources with selective insecticides could be especially effective. However, substantial knowledge gaps remain regarding effective bait formulations, active ingredients, application timing, spatial deployment, and potential impacts on non-target organisms. The development of selective toxic baits could substantially reduce reliance on cover sprays of broad-spectrum products and reduce the presence of pesticide residues in figs.

Current evidence suggests that effective biological control of *S. adipata* is most likely to rely on the conservation and enhancement of generalist natural enemies, particularly ants and ground-dwelling beetles that attack larvae and pupae in the soil. Classical parasitoid-based control appears limited, as larval parasitism is likely to be constrained by the protective role of fig latex, and no consistent field parasitism has been documented. In contrast, entomopathogenic fungi and nematodes emerge as the most promising biological control agents for inundative control, given their demonstrated efficacy against tephritid flies and their ability to infect soil-dwelling stages. Targeting pupae through soil applications or auto-inoculation + horizontal transmission strategies may therefore represent a key intervention. Future research should prioritize field-based evaluations of these pathogens and their integration with predator-compatible management practices.

Orchard sanitation remains a cornerstone of *S. adipata* management. Frequent removal and destruction of infested figs are essential to prevent rapid population growth and local reinfestation. Beyond figs removal, careful handling and movement of harvested figs and collection materials are critical, as these can inadvertently facilitate pest spread within and between production areas. At a broader scale, the movement of figs and plant material represents a significant phytosanitary risk and must be strictly controlled in regions where *S. adipata* is absent. Preventing the introduction of this pest into new areas through effective regulatory measures is therefore of paramount importance.

Continued research addressing these key areas will be essential to develop robust, sustainable, and effective management strategies capable of mitigating the growing impact of this serious pest on fig production in its native region and in production systems elsewhere in the world.

## Data Availability

No new data were created or analyzed in this study. Data sharing is not applicable to this article.
